# Extraction of Teeth in Pregnant Women

**Published:** 1882-11

**Authors:** 


### ARICLE IV.

### *Extraction of Teeth in Pregnant Women.*

From a report of the proceedings of the St. Louis Medical Society, published in the *St. Louis Medical and Surgical Journal*, we extract the following:

Dr. Borck.—I would suggest a question. Is it advisable to allow the extraction of a tooth, or of teeth in a patient who is pregnant? Several times I have been asked this question by dentists. Some eminent dentists are afraid to extract an aching tooth because the patient is pregnant. They are afraid of producing an abortion.

Dr. Green.—The extraction of a tooth in a pregnant woman does not necessarily produce an abortion. Of course there may be instances where such an effect would follow, but if the woman is suffering, and we cannot relieve her by any other means, I would recommend the extraction of the tooth.

Dr. McPeeters.—There is a form of tooth-ache which is sometimes a symptom of pregnancy. The teeth are sound, and of course it would do no good to extract them.

I should not hesitate to advise the extraction of a carious tooth, but in case of this neuralgia which sometimes accompanies pregnancy, I should advise against it, because we sacrifice the tooth uselessly.

Dr. Hughes.—I don't suppose we can adopt any one rule that will apply to all kinds of patients. Some patients when pregnant, are extremely hyperæsthetic; the hyperæsthesia extends to the branches of the fifth pair; other women, who are more or less nervous when not carrying a child, seem to possess more nerve then than at any other time; they seem to be in a better condition. I don't know in view of the varying and variable physiological condition in which we find women in the pregnant state, that we could arrive at any definite rule applicable to all cases. I suppose, however, that Dr. Borck has reference to the propriety of extracting decayed teeth—teeth which are themselves the peripheral source of great central irritation. Of course no one would think of taking out sound teeth to relieve a neuralgic condition. The question, ought a tooth to be extracted from a pregnant woman, is not a question upon which we could ever arrive at any definite conclusion which would embody a rule, and therefore it would be fruitless to engage in its discussion. If the question is asked, under what circumstances ought the removal of a tooth to be advised during pregnancy, you have a question that might be discussed. It is simply a question of individual temperaments, of conditions of the patient, and of the existence of centric or excentric irritation; the existence or non-existence of central or peripheral irritation. And, if a pregnant woman is extremely hyperæsthetic, and you can find a focus of origin for it in the peripheral irritation of a decayed tooth, there would be no impropriety, in the majorty of cases, I apprehend, in the removal of that decayed tooth. If in a condition of general nervous excitation, especially if centered in the brain or cord, you have any form of spasmodic display, and you find a possible peripheral sources of the irritation, I think the general sentiment of the profession would concur in the propriety of removing that possible scource of peripheral irritation. That is all there is to that question.

Dr. Jonaston.—Some years ago I was called to a lady who had been married six or eight weeks. The second left molar was decayed and an abscess was forming, and protruded from the root of the tooth. The abscess was painful, and I advised opening it. She consented, and I took my lancet and opened the abscess. This produced a tremendous shock, and in twenty-four hours she aborted. This case occuring in my early practice, has made me very careful about extracting a tooth from a patient during the early part of pregnancy, if she were of a nervous temperament; it is a hazardous practice. But if the tooth-ache continues, the reflex irritation of the pneumogastric nerve, connecting with the great sympathetic, may induce uterine contraction, and cause the woman to abort. In such a case we should recommend that the tooth be pulled. There is no rule in the practice of medicine, and no rule as regards drugs, except castor oil. I have given calomel for twenty years under the supposition that it acted on the liver, and now we are told that it doesn't act upon the liver at all.

Dr. Hurt.—I think we are all obliged to concede the possibility of the extraction of a tooth during pregnancy producing abortion under certain conditions. There is no doubt, also, that there are circumstances under which the extraction of a tooth during pregnancy ought to be advised. The loss of a sound tooth ought not to be allowed unless something is going to be accomplished by it that cannot be accomplished otherwise. But I would have no hesitation about advising the extraction of a tooth from a pregnant woman if it was absolutely necessary to relieve her from a distressing, harassing pain that was wearing her out; and in doing this we may administer an anæsthetic without interfering in the least with the pregnancy. When she is under chloroform or ether, we obviate the shock which is usually attendant upon the extraction of teeth. And experience has taught that pregnant women are very tolerant of these agents.

---

In writing to the *Journal of the British Dental Association*, Mr. Alfred Coleman says:—"*Apropos* of the interesting question raised by Mr. Sewill at the meeting of the Odontological Society, viz. 'The removal of teeth, under nitrous oxide, in advanced pregnancy,' I think the following case may be of interest to your readers.

A lady from Oxfordshire visited me on the 14th day of March last. She had suffered excruciating pain in the lower third molar of the left side, which she wished removed under gas, but I was informed by her husband that she expected to be confined in a fortnight. Now I was aware that this lady had been previously four or five times confined at the full period, but had never given birth to a living child; still as I entertained the views expressed by Mr. Sewill, that it was far preferable for the patient to have the tooth removed under gas than suffer the pain, which I have known to bring on a miscarriage, I willingly consented to its being administered to her, and removed the tooth.

On the 2d of this month I was pleased to hear from her own lips that on the 1st of April last she had for the first time given birth to a living child—a son. She also told me on this occasion that she came up to town without saying a word to her medical attendant, as she felt almost sure he would object to her taking gas, but on that matter she relied more upon my opinion.

As my patients are people of considerable property, this this is a gratifying circumstance."

And the same journal says:—The following extract will be read with interest. It is an able summary of the evidence bearing upon maternal impressions, and it supports the view expressed at this meeting, namely, that the popular notions on this matter are not based upon science. We take the excerpt from the just published admirable work of Mr. Noble Smith on the "Surgery of Deformities." "This book will well repay the perusal of those specially working at the subject of deformities of the jaws. Although it does not deal specifically with this topic, there are many points connected with the origin and pathology of deformities generally, which have a connection with those of the jaws and neighborhood.

"MATERNAL IMPRESSIONS.—The belief that a maternal impression can produce deformity *in accordance with the idea of the mother* has a very acient origin, and is entertained by many medical men at the present time. It therefore becomes necessary to state the following facts, which are in opposition to such belief:

1. That the resemblance of the deformity to the object which has impressed the mother is generally an imaginary one.

2. That the maternal impression is almost invariably only alluded to *after* the discovery of the deformity.

3. That the ovum as soon as it leaves the ovary, ceases to be connected, either by the nervous or vascular systems, with the mother, and therefore, in resembling very closely the egg of an oviparous animal, becomes almost equally unlikely to be influenced in the manner referred to.

4. That from the nature of the deformity we may usually know that the error in formation must have occurred at a much earlier period than the date of the supposed cause, and this is especially the case when the error is one of excess.

5. That, in the words of Dr. Blundell, 'it is contrary to experience, reason, anatomy, to believe that the strong attention of the mother's mind to a determinate object or event' can cause 'a specific impression upon the child without any injury from without.'

A great shock to the mother, even if only a maternal one, may effect the general condition of the fœtus, may retard its development, or may even cause its death; but that the mother's mind being affected by the sight of a deformed or injured hand, lip, or other portion of the body can produce a malformation *of a corresponding part of the body* of her child *in utero* is a supposition which physiological facts will not allow us to entertain.

If it be thought necessary to pursue this subject further, the only manner in which reliable evidence can be obtained is as follows:—The observer must question each mother *before delivery* as to any impressions she may have formed upon the subject, and compare these with the actual condition of the child when born. It is probable that nearly every pregnant woman entertains doubts and fears with regard to the condition of her coming offspring, and that when her child is born in a normal condition all apprehensions are soon forgotten, but that when an abnormity exists the fear is magnified into an important fact.

In past ages congenital deformities were attributed to various causes, amongst which may be mentioned Divine vengeance, witchcraft, intercourse with animals, &c.; but the knowledge which we now posess of the various phenomena of embryonic development enables us to determine that many deformities depend either upon *arrest* or *excess* of the processes of formation."—*Dental Advertiser.*

---

